# Comparison of bacterial communities from lava cave microbial mats to overlying surface soils from Lava Beds National Monument, USA

**DOI:** 10.1371/journal.pone.0169339

**Published:** 2017-02-15

**Authors:** Kathleen H. Lavoie, Ara S. Winter, Kaitlyn J. H. Read, Evan M. Hughes, Michael N. Spilde, Diana E. Northup

**Affiliations:** 1 Biology, State University of New York, College at Plattsburgh, Plattsburgh, NY, United States of America; 2 Biology, University of New Mexico, Albuquerque, NM, United States of America; 3 Institute of Meteoritics, University of New Mexico, Albuquerque, NM, United States of America; Free University of Bozen/Bolzano, ITALY

## Abstract

Subsurface habitats harbor novel diversity that has received little attention until recently. Accessible subsurface habitats include lava caves around the world that often support extensive microbial mats on ceilings and walls in a range of colors. Little is known about lava cave microbial diversity and how these subsurface mats differ from microbial communities in overlying surface soils. To investigate these differences, we analyzed bacterial 16S rDNA from 454 pyrosequencing from three colors of microbial mats (tan, white, and yellow) from seven lava caves in Lava Beds National Monument, CA, USA, and compared them with surface soil overlying each cave. The same phyla were represented in both surface soils and cave microbial mats, but the overlap in shared OTUs (operational taxonomic unit) was only 11.2%. Number of entrances per cave and temperature contributed to observed differences in diversity. In terms of species richness, diversity by mat color differed, but not significantly. *Actinobacteria* dominated in all cave samples, with 39% from caves and 21% from surface soils. *Proteobacteria* made up 30% of phyla from caves and 36% from surface soil. Other major phyla in caves were *Nitrospirae* (7%) followed by minor phyla (7%), compared to surface soils with *Bacteroidetes* (8%) and minor phyla (8%). Many of the most abundant sequences could not be identified to genus, indicating a high degree of novelty. Surface soil samples had more OTUs and greater diversity indices than cave samples. Although surface soil microbes immigrate into underlying caves, the environment selects for microbes able to live in the cave habitats, resulting in very different cave microbial communities. This study is the first comprehensive comparison of bacterial communities in lava caves with the overlying soil community.

## Introduction

Most life on Earth in the aphotic subsurface is microbial [1], but there is much that we do not yet know about subsurface life. Caves can provide a natural way to access subsurface environments ranging from very deep limestone caves (Krubera Cave in the Western Caucasus is more than 2,190 m deep [2]), to shallow caves, such as lava caves that have an overburden of up to 10 m [3]. Discovery of extensive lava flows and lava caves on Mars [4] supports the concept that Earth’s lava caves may serve as a model for the study of life on other planets (astrobiology) [5,6,7].

**Fig 1:**
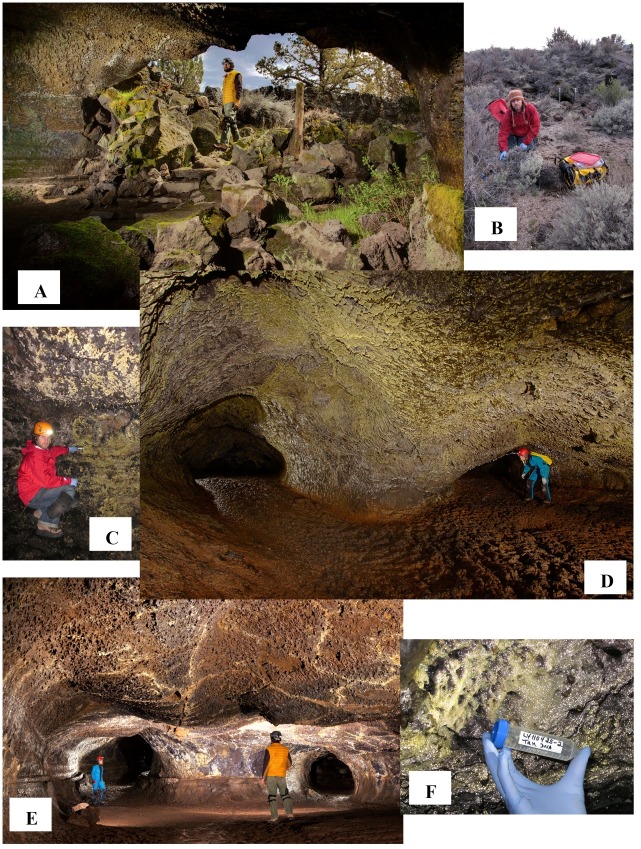
Overview of some of the lava cave sampling sites and caves, plus a view of the surface terrain at Lava Beds National Monument, CA (LABE). (A) Entrance to Valentine Cave. (B) Surface samples taken above Hopkins Chocolate Cave. (C) Yellow microbial mat sampling site in Valentine Cave. (D) Extensive yellow microbial mats on walls of Hopkins Chocolate Cave. (E) Passage in Valentine Cave showing less microbial mat coverage near the entrance. (F) Tan microbial mat sample taken in L-V460 Cave. Photos copyright Kenneth Ingham (A, D, E) and Diana Northup (B, C, F).

**Fig 2:**
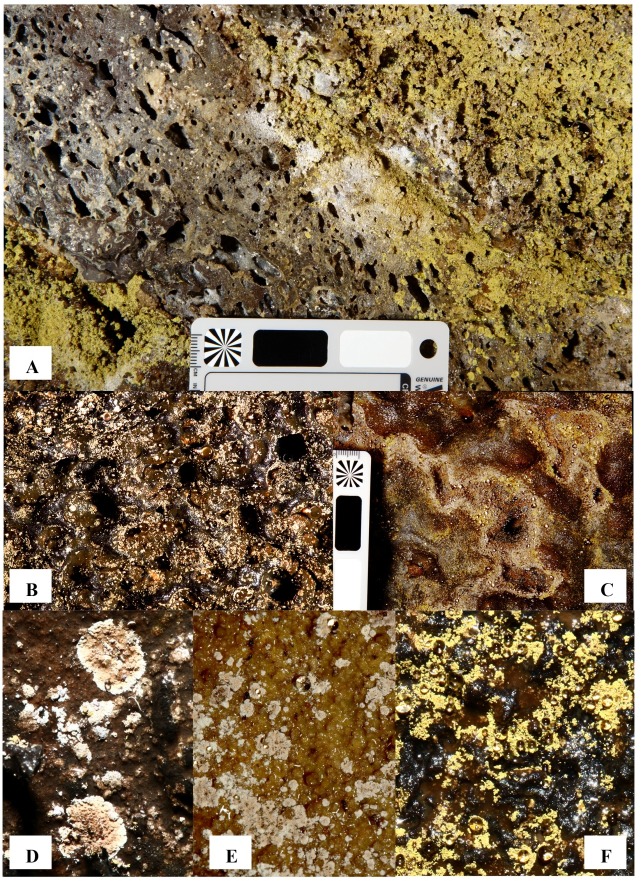
Microbial mat and colony morphology. (A) Overview of predominantly yellow and white microbial mats, some separate and some intermixed. (B) Overview of tan and white microbial mats. (C) Intermixed tan, white, and yellow microbial mats. Close ups of (D) tan and white colony morphology, (E) tan colonies, and (F) yellow colonies.

Lava caves, formed during active lava flows, contain diverse microbial mats that range from extensive mats covering walls and ceilings to small, scattered colonies (Fig 1 and Fig 2). Mat colors include white, yellow, tan, gold, orange, and pink, with shades in between [7–11]. Despite their extensive nature, little is known about microbial mat diversity (reviewed in [11,12]). Studies of microbial diversity [7–15] in lava caves lag behind such studies in karst caves. Stoner and Howarth [16] first described the mats or “slimes” in Hawaiian lava caves using culture-dependent methods for isolation of chemoheterotrophic microorganisms and reported on the presence of fungi and aerobic bacteria. They suggested that white and brown slimes might be important sites for nutrient cycling in caves, particularly nitrogen.

Lava caves are extreme environments, simplified by the lack of photosynthesis in the deep or dark zone of the cave, resulting in extremely oligotrophic conditions. The simplified nature of caves makes them a model natural laboratory to study factors controlling biological diversification [17,18]. The isolation of most caves limits the ability of organisms to migrate, resulting in high levels of endemism among troglobionts and stygobionts as the norm [19]. Culver et al. [20] found that about 30% of cave-adapted invertebrate species in U.S. caves are found in only a single cave. The results of Hathaway et al. [21] show that the trend can be extended to bacterial diversity in Azorean and Hawaiian lava cave microbial mats. The authors were far from sampling total diversity, but less than 5% of the OTUs (operational taxonomic unit) found in lava caves occur in other caves or in other volcanic environments. Sequences were more likely to be related to samples from the same cave or the same island than between islands. If microbial distribution is ubiquitous, then they would expect a higher percentage of shared OTUs between the two island archipelagos.

Biospeleologists originally thought that cave microbes were simply a subset of surface microbes washed into underlying caves [22,23]. Ortiz et al. [24,25] recently published what we believe to be the first comparisons of cave microbial diversity with the overlying soil microbial community. Their studies focused on bacterial diversity across carbonate speleothem surfaces sampled by swabbing from a karst cave, Kartchner Cavern in Arizona. Comparison of bacterial taxonomic profiles to surface soil samples revealed major differences and only a 16% overlap between cave speleothem and surface soil OTUs [24].

Our study is the first and most comprehensive to compare lava cave bacterial mat communities to bacterial communities from the overlying surface soil of each cave. We examined a range of environmental, geographical, and chemical factors that may contribute to bacterial diversity in microbial mats of different colors (tan, white, and yellow) from lava caves in Lava Beds National Monument, California, USA.

## Materials and methods

### Field studies ethics statement

All sampling was done under Permit LABE-2011-SCI-0007 issued to Northup by the National Park Service (Lava Beds National Monument, 1 Indian Well Headquarters, PO Box 1240, Tulelake, CA 96134). Lava Beds National Monument is a federally-protected area under the National Park Service, Department of the Interior. No protected species were sampled. Shawn Thomas, Shane Fryer, and Joke Vansweevelt have given their consent to have photos of themselves, taken during sampling trips, used in this manuscript.

### Sampling sites

Lava Beds National Monument (LABE) is located in northern California close to the borders of Oregon and Nevada [26]. The Monument covers 190 km^2^ on the NE flank of the Medicine Lake Volcano. Two-thirds of the lava came from the Mammoth and Modoc craters over the last two million plus years and as recently as 1,100 years ago. Flows are largely of basalt with smaller amounts of silica-rich basaltic andesite [27].

LABE has the largest number of lava caves in North America, with 778 known [26]. Twenty-five of the lava caves have signed entrances and developed trails for ease of visitation. The area is a high-elevation (1219–1737 m above sea level), semi-arid desert with average yearly precipitation of 375 mm. Temperature ranges from an average low of -5.4°C in January to an average high of 22.3°C in July and August. Some of the lava caves contain perennial ice [27].

We worked with LABE personnel to select seven lava caves to cover a range of parameters (Table 1): amount of human visitation, age of the lava flow, surface vegetation, elevation, number of entrances, branching complexity, and mapped length of the caveIn addition to the cave samples, a sample of soil overlying each cave entrance (e.g. Fig 1B) was collected for comparison and photo-documented at the collection site (Fig 1F).

**Table 1:** Characteristics of the seven study cave sites in LABE.

Cave	Surface Soil	Visitation	Lava Age	Surface	Elevation	# Entr	Branches (Nodes)	Length
**Catacombs**	C15	High	36,000 ±16,000	Sagebrush	1,524 m	1	32	2571 m
**Golden Dome**	GD25	High	36,000 ±16,000	Sagebrush	1,378 m	2	7	679 m
**GE-L350**	GE13	Low	16,000	Sagebrush	1,506 m	1	9	439 m
**Hopkins Chocolate (HCC)**	HC15	High	36,000 ±16,000	Sagebrush	1,506 m	4	4	519 m
**L-V460**	L14	Low	12,260	Juniper	1,360 m	2	5	700 m
**S-L280**	S10	Low	36,000 ±16,000	Sagebrush	1,372 m	2	5	671 m
**Valentine**	V16	High	12,260	Juniper	1,3768m	1	12	651 m

Cave name or code for backcountry caves. Visitation = number of visitors, grouped into High (open to the public) and Low (closed to visitation). Lava Age = Age of lava flow. Surface = surface vegetation, where Sagebrush = Big sagebrush-antelope bitterbrush scrubland and Juniper = Juniper-mountain mahogany scrubland. Elevation is the elevation in m at the entrance a.s.l. # Entr = number of accessible or functional entrances into the cave. Branches (Nodes) is an indication of the geometric complexity of the cave by counting the number of passage branch points. Length is the mapped length of the cave in m.

Cave samples (Table 2) were characterized by color of the microbial mats (tan, white, and yellow e.g. Fig 2), distance from the nearest entrance, temperature, humidity, and RH, and pH when suitable water pools or dripping water were available near mats. All samples were photo-documented at the collection site

**Table 2:** Cave samples bymat color and environmental characteristics in the cave.

Cave	Sample	Color	Distance from entrance	T° C	%RH	pH
**Catacombs**	C1	T	51 m	13.6	61	nd
**Catacombs**	C2	Y	51 m	13.6	61	nd
**Catacombs**	C9	W	76 m	15.7	45	nd
**Golden Dome**	GD1	Y	278 m	9.3	99	8.13
**Golden Dome**	GD2	T	276.5 m	9.2	100	7.96
**Golden Dome**	GD3	W	276.5 m	9.2	100	7.96
**Golden Dome**	GD16	Y	278 m	9.3	99	8.13
**GE-L350**	GE1	T	64 m	9.9	87	7.45
**GE-L350**	GE2	Y	64 m	9.9	87	7.45
**GE-L350**	GEM2	W	67 m	7.1	86.6	nd
**HCC**	HC1	Y	37 m	11.5	89	nd
**HCC**	HC6	T	107 m	8.9	100	7.98
**HCC**	HC7	Y	107 m	8.9	100	7.98
**HCC**	HCC1	W	105 m	8.9	100	7.97
**L-V460**	L1	Y	200 m	9.3	96.3	7.31
**L-V460**	L3	W	200 m	9.3	96.3	7.31
**L-V460**	L9	T	87 m	9.8	92.7	nd
**S-L280**	S2	Y	255 m	16.1	82.1	nd
**S-L280**	SC2	W	135 m	16.1	82.1	nd
**S-L280**	S4	T	145 m	13.1	70.6	nd
**Valentine**	V1	Y	135 m	11.2	100	7.45
**Valentine**	V2	T	135 m	11.2	100	7.45
**Valentine**	V13	W	170 m	11.3	100	7.75

C = Catacombs; GE and GEM = G-L350; GD = Golden Dome; HC and HCC = Hopkins Chocolate; L = L-V460; S and SC = S-L280; and V = Valentine. Color: T = Tan, Y = Yellow, W = White. Distance is the distance in m from the nearest entrance to the sampling site. Temperature, RH, and pH data were collected in April 2011 and Aug 2012. (nd = not determined)

### Temperature, RH, and pH measurements

Temperature (web bulb and dry bulb in order to obtain an approximate RH) was taken in April, 2011 and in September, 2012 with an IMC temperature probe (http://www.imcinstruments.com/), which was calibrated at frequent intervals in the cave to improve accuracy. Wet bulb readings were obtained with the IMC probe sheathed with wicking soaked in deionized water before each reading. For some RH samples a portable Kestrel 3000 wind meter (https://kestrelmeters.com/products/kestrel-3000-wind-meter) was used, which was calibrated at the beginning of each cave. A Javascript program (http://home.fuse.net/clymer/water/wet.html) used dry and wet bulb temperatures to approximate relative humidity. Readings for pH were taken with a Twin Cardy pH meter (Spectrum Technologies, Inc., http://www.specmeters.com/nutrient-management/ph-and-ec-meters/ph/cardy-twin-ph-meter/), calibrated with pH 7 buffer.

### Sample collections for DNA and Scanning Electron Microscopy (SEM)

Sampling took place in April 2011 with additional sampling (GEM2, HCC1, SC2) in August 2012 to increase the number of white mat samples. Yellow, white, and tan mats were sampled from each cave. Samples for DNA extraction were collected aseptically with a flame-sterilized cold chisel into a sterile 50 cc Falcon tube. Soil samples were collected from above each cave entrance by removing any surface plant detritus and scooping the top 2 cm of soil into a sterile 50 cc Falcon tube. All samples for DNA were covered within hours with sucrose lysis buffer [28] to release and stabilize the DNA. All samples were brought to the Northup Lab at the University of New Mexico for further processing and analysis within seven days. We collected surface soils from above seven caves, and had seven samples of white and tan mats and nine samples from yellow mats, with at least one of each color from each cave.

Samples for scanning electron microscopy (SEM) from rock chips were mounted directly onto SEM stubs with super glue and placed in a carrying case for transport. A microbial mat sample (L-V460-110425-6), consisting of chips of the wall rock with white to pale yellow colonies, was taken approximately 200 m into Cave L-V460 at the bottom of a pillar. One of the two yellow microbial mat samples (HC110423-5) analyzed with SEM was a rock chip with yellow colonies from the floor of Hopkins Chocolate Cave, approximately 36 m into the cave. The second yellow mat sample (S-L280-110427-3) was taken approximately 60 m into Cave S-L280 and 1 m above the floor and 1 m below the ceiling.

### Water chemistry analysis

Samples for chemical analysis were preserved with 6N hydrochloric acid in the field, as described in [29]. Amounts of chloride, nitrite, nitrate, phosphate, and sulfate were analyzed using a Dionex Ion Chromatograph DX-100 (Dionex, Sunnyvale, CA, USA) as described [29].

### Molecular phylogeny

#### DNA extraction, sequencing, and sequence analysis

DNA was extracted from triplicate samples of rock chips with microbial mats from each cave by mat color and from the surface soil samples using the MoBio Power Soil^TM^ DNA extraction kit following manufacturer’s protocol except we used bead beating rather than vortexing, which the Northup Lab finds to be more effective at releasing DNA from Gram positive cells.

#### Polymerase Chain Reaction (PCR)

PCR was performed to verify the quality and quantity of the DNA prior to sequencing. One hundred twenty five to three hundred ng of purified DNA was used to amplify the 16S rRNA gene from environmental DNA by PCR with universal primers, p46 forward (5’-GCYTAAYACATGCAAGTCG-3’) and p1409 reverse (5’-GTGACGGGRGTGGTGTRCAA-3’; [30] and AmpliTaq LD (Applied Biosystems) with an MJ thermal cycler using: 4 min denaturation at 94°C followed by 35 cycles of 45 sec annealing at 55°C, extension for 2 min at 72°C, denaturation for 30 sec at 94°C, with a final 45 sec 55°C and a 20 min 72°C extension step after cycling was complete.

#### Sequencing and phylogenetic analysis

Samples were analyzed with next-generation sequencing of the 16S SSU gene bacterial V1-3 region (primer 27F) using Roche FLX and Titanium 454 technology conducted by MR DNA, Shallowater, TX (http://www.mrdnalab.com/).

All 454 data were processed in QIIME 1.9.1 [31]. Quality control and trimming of the 454 dataset were done using the split_libraries.py command with a lower length (-l) of 100 bp and an upper length (-L) of 500. A quality score (-s) of 30 was chosen. Removal of erroneous sequences (denoising) and OTU clustering were done using pick_de_novo_otus.py pipeline with the sumaclust option [32]. The sumaclust algorithm is mainly useful to detect the 'erroneous' sequences created during amplification and sequencing protocols. OTUs were clustered at the 97% similarity level using sumaclust. The pick_de_novo command also picks the representative set and assigns taxonomy using uclust [33] against the greengenes 13.8 database [34]. The pipeline also aligns and builds a phylogenetic tree using pynast [35] and fasttree [36] from the representative sequence set. Chimera checking was done using USEARCH to detect artifacts created during sequencing. Good’s coverage showed that we were successful in getting nearly all of the diversity from our samples. Values ranged from 99.11% to 87.13% with an average value of 94.98%. Library sizes ranged from 471 to 4317. Rarefaction curves (S1 Fig) were generated using a custom function written by Bela Hausmann (https://github.com/joey711/phyloseq/issues/143) and is also available in our zenodo archive. These plots compare estimated total richness per sample at a given depth. The rarefaction curves are calculated with observed (total richness), Shannon, Chao1 and their associated uncertainties.

Diversity by phyla with the *Proteobacteria* separated out by class was compared for all samples. The L2 phyla data were reduced to eighteen groups, including unassigned phyla and a group we called minor phyla. The minor phyla are entries that had less than 1000 OTUs across all samples. Of a total of 140,848 OTUs, 26,609 were from the surface samples, and 38,214 from tan, 36,014 from white, and 40,011 from yellow mats. The process was repeated at the L6 genus level resulting in nine groups, an unassigned group, and minor genera with 471 taxa.

Sequences submitted to the NCBI GenBank database (www.ncbi.nlm.nih.gov/genbank/) were assigned Accession Numbers JX694094-JX702544, and the three additional white samples KP705489-KP706447.

### Statistical analysis

Community dissimilarity was visualized using the phyloseq package [37] and ggplot2 [38] in R [39]. Alpha diversity was analyzed using observed OTUs, Shannon and Chao1 indices in the phyloseq package. Alpha diversity measures were carried out on the raw data as recommended [37]. Observed richness will scale with increasing library size; however both Chao1 and Shannon are robust measures of richness and diversity, which account for differences in library size (rarefaction curves in S1 Fig). Observed OTUs is the raw number of OTUs present in each sample of quality controlled and clustered sequences as described above. Beta diversity was analyzed using non-metric dimensional scaling (NMDS) with the Brays-Curtis distance using the vegan package [40] in R. The Brays-Curtis distance was picked because it is invariant to changes in units, unaffected by additions and removal of samples, and NMDS recognizes differences in total abundances when relative abundances are similar. The ordination of the samples was done using custom R scripts by Umer Zeeshan Ijaz available at: http://userweb.eng.gla.ac.uk/umer.ijaz/bioinformatics/ecological.html.

Differential abundance of taxa was characterized using the DESeq2 package [41] with parameters fitType = “local”. An adjusted p-value threshold of 0.1 was used to calculate log2 fold changes between surface soils and cave microbial mats.

Phylogenetic tree analysis was carried out in the phyloseq package. The data were subset by phyla that were differentially abundant as determined by DeSeq2. Tree files with tips label were written out using write.tree(phy_tree(phyloseq_obj),file = "phylum_name.newick"). The tree, tip labels, and traits (cave or surface) were loaded into Interactive Tree of Life v3 (http://itol.embl.de/) [42] for visualization. Traits were assigned to the tree tips as relative abundance of the OTUs present in the cave.

Total bacterial richness for cave samples was tested using a hierarchal generalized mixed effects model in R with the stanarm [43] package. Richness data were normalized in QIIME before modeling using normalize_table.py -i lavabeds.biom -a DESeq2 -o DESeq2_normalized_otu_table.biom–DESeq_negatives_to_zero. Cave, color of mat, visitation, and cave geometry were assigned as random effects (levels of hierarchy). The following options were used with stan_glmer: family = gaussian, dapt_delta = 0.95, prior = normal(location = 0, scale = 8), seed = SEED, iter = 50000, cores = 6.

## Results

### Water chemistry

Chemical analysis of water samples collected from each cave with available standing water is shown in Table 3. Chlorine levels averaged (5.086 ppm), slightly higher than the EPA Maximum Contaminant Level Goals for drinking water of 4 ppm [44]. EPA levels for nitrate (1 ppm) and nitrate (10 ppm) were not exceeded in most cave water samples. There are no phosphate level standards set by the EPA, but our cave samples were all low. Sulfate water standards are 250 ppm, and ours were all very low.

**Table 3:** Water chemistry of cave water samples from Lava Beds National Monument. See Table 1 for the cave sample abbreviations. Bromide (not shown) all below detection limits.

Cave sample	Chloride (Cl mg/L)	Nitrite (NO_2_ mg/L)	Nitrate (NO_3_ mg/L)	Phosphate (PO_4_ mg/L)	Sulfate (SO_4_ mg/L)
CAT7	12.571	nd	0.6039	nd	0.6103
CAT13	2.184	0.9866	0.5924	nd	0.3251
GD20	3.567	0.5526	0.7286	nd	nd
GE26	9.203	nd	0.8325	nd	0.4704
GE10	5.128	0.3320	0.9140	nd	0.4329
HC3	2.116	nd	0.6606	0.6715	0.5391
HC10	1.821	nd	1.5608	nd	1.1231
L-V460-4	4.493	0.5778	2.7485	0.5371	0.5851
L-V460-12	2.031	nd	0.7116	0.5571	0.5505
S-L280-6	3.702	0.4489	8.0036	0.4697	2.3381
VAL3	9.132	0.3113	0.5483	nd	0.5910

nd = not detected

### Surface soil versus cave alpha diversity

**Fig 3:**
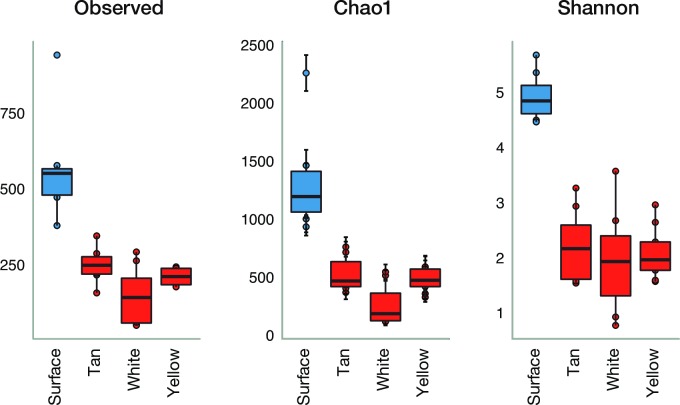
Alpha diversity indices (richness, Chao1, and Shannon). Box plots of surface soils and cave microbial mats by color. Surface soil samples are in blue and cave samples in red.

Measures of alpha diversity among surface soil samples and cave samples by color of mat (Fig 3) show species richness (Observed and Chao1 [45]) and relative abundance (Shannon and Simpson’s). Simpson is less influenced by singletons (i.e. rare taxa) than Shannon’s Index, but Shannon’s in less sensitive to difference in library size. The means are very different, with much greater bacterial diversity in soil than cave. Soil bacterial diversity is more evenly distributed among OTUs. Cave samples are less evenly distributed, with a wide distribution of Simpson’s values with many outliers in contrast to soil samples. In-cave variation is much higher than surface soil variation. In terms of species richness in differently colored mats, tan samples have higher diversity, followed by yellow and then white. In terms of Shannon’s and Simpson’s indices, the three colors of microbial mats are not that different. Fig 3 shows the difference between cave microbial mats and surface soils in respect to total richness of each sample (observed OTUs) and Shannon Index. In all cases surface soils have more OTUs and higher Shannon indices indicating higher diversity. Rarefaction curves compare estimated total richness per sample at a given depth and are included in the supplemental information (S1 Fig). The rarefaction curves show a linear relationship between sampling depth and species richness. The Chao1 estimated richness and Shannon level off after 1000 and 500, respectively.

### Taxonomy by phylum and proteobacteria class

**Fig 4:**
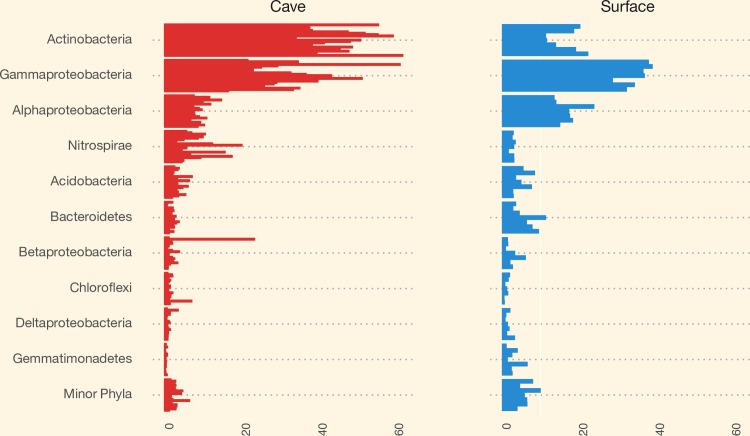
Staggered bar chart of relative abundance of OTUs. Major and minor phyla by all cave samples and all surface samples. Cave samples have a greater relative abundance of *Actinobacteria* and *Nitrospirae*.

Composition by the top five phyla and four proteobacterial classes, plus minor phyla based on OTUs, are presented in Fig 4. Comparisons of surface soil samples with the underlying cave samples show some differences. Most notable is the lower percentage of *Actinobacteria* in surface soil samples (21%) versus cave samples (39%), the reduction in the *Nitrospirae* in surface (3%) vs. cave samples (7%), and the increase in *Alphaproteobacteria* in the surface soil samples (17%) compared to the cave samples (10%). Smaller, but significant differences that decrease from surface to cave are seen with the *Bacteroidetes* (surface 8% and cave 2%); *Gemmatimonadetes* (surface 3% and cave <1%); and *Planctomycetes* (surface 2% and cave 1%). The *Gammaproteobacteria* increase between surface (18%) and cave (20%), while *Betaproteobacteria* (surface 4% and cave 3%) and *Deltaproteobacteria* (surface 4% and cave 1%) both decline in cave samples. None of the phyla differed significantly by mat color. Some differences were observed by mat color in the *Actinobacteria* (tan: 37%, white: 39%, yellow: 44%), *Gammaproteobacteria* (tan: 19%, white: 24%, yellow: 20%), *Nitrospirae* (tan: 11%, white: 4%, yellow: 7%), and *Betaproteobacteria* (tan: 3%, white: 6%, yellow: 2%).

### Molecular phylogeny by family or genus

**Fig 5:**
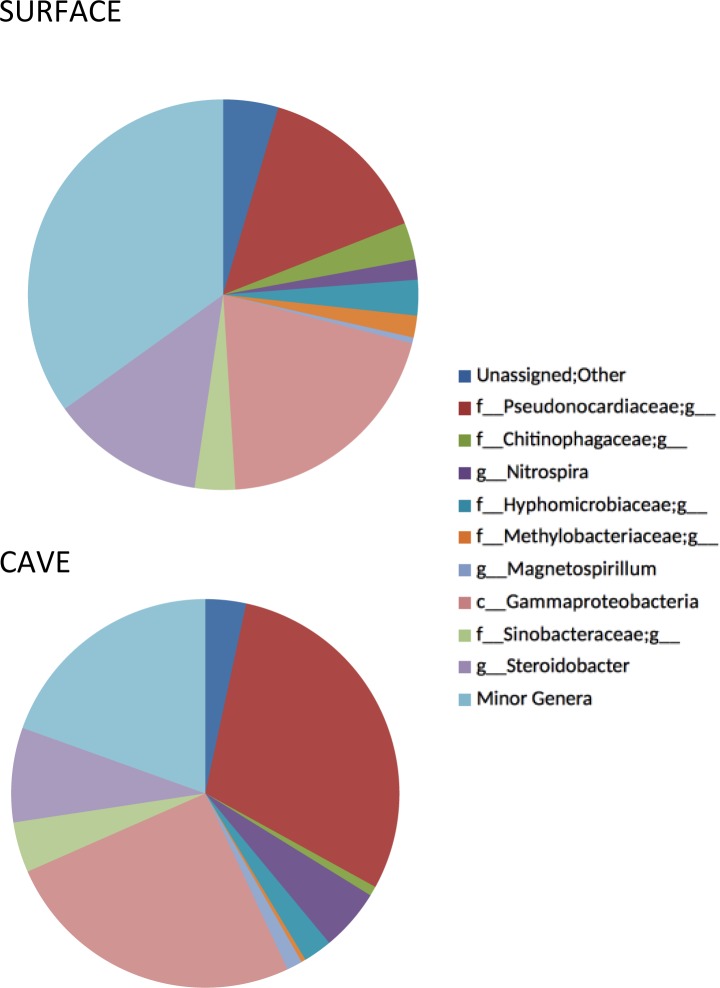
Percent OTUs by lowest level of classification (family or genus). (A) mean surface soils and (B)mean cave microbial mats.

The percentage of OTUs by genus or family is presented in Fig 5 for surface soil and cave mat samples. Results from the analysis of diversity by genera are interesting in several respects. First of all, a large percentage of OTUs are novel. Of the nine major genera, only four are identified to genus level, four to family, and one to class. There is also a group called unassigned, which could not even be classified to bacteria. These unassigned OTUs may represent archaeal DNA that amplified with our primers or may be organisms that are particularly difficult to classify through 454 sequencing, like members of the *Verrucomicrobia*. Bergmann et al. [46] used barcoded pyrosequencing with surface and subsurface soils to reduce primer bias, and found that *Verrucomicrobia* are ubiquitous and were often the dominant phylum in their samples. In our study, no individual *Verrucomicrobia* genus met our criteria for inclusion in Fig 5.

A major difference between surface and cave samples is the proportion of the family *Pseudonocardiacae* present with 14% of surface soil OTUs vs. 30% of cave OTUs. Other major genus level OTUs fell within the *Acidobacteria*, *Nitrospiraceae*, and *Bacteroidetes*. Within the *Acidobacteria* there are three groups, all belonging to the candidate class *Chloroacidobacteria*. There are two orders *PK29* and *PK10* that are not identified further. The *Nitrospiraceae* is classified to the genus *Nitrospira*. The *Bacteroidetes* group is identified to the family *Chitinophagaceae*, which are elevated in soils where insect chitin is higher than in the cave, a defining characteristic of this family [47], although new isolates from oligotrophic lake waters lacked this ability [48].

The remaining major bacterial OTU groups are all members of the *Proteobacteria*, with three from the *Alphaproteobacteria* and three *Gammaproteobacteria*. The *Alphaproteobacteria* OTUs are two members of the order *Rhizospiriales*, one family *Hyphomicrobiaceae* and one *Methylobacteriaceae*, and one *Rhodospirillaes* identified to the genus *Magnetospirillum*. Together the *Rhizospiriales* account for only 2% of cave species, but 5% of surface species, probably associated with the rhizosphere. The *Gammaproteobacteria* include the class *Gammaproteobacteria* and two members of the *Sinobacteraceae* with one family, *Sinobacteraceae*, and the genus *Steroidobacteria*. The type species is *Steroidobacteria denitricans*, a nitrate oxidizer [49], and the family *Sinobacteraceae* contains only two described species.

### Dissimilarity among surface soils and cave microbial mats

**Fig 6:**
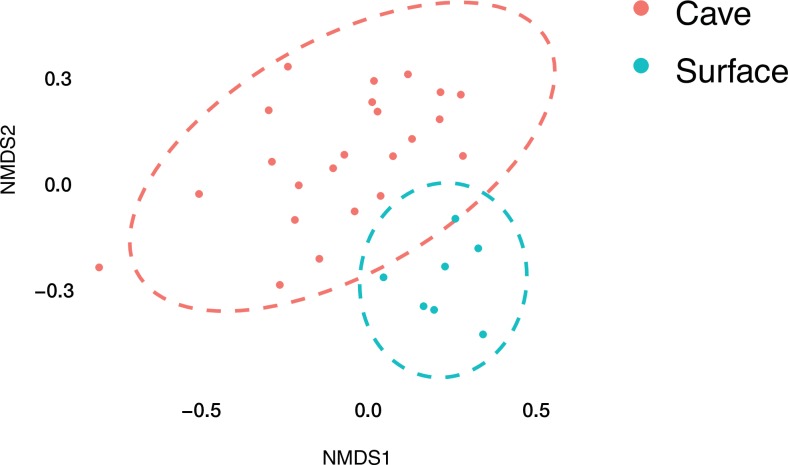
NMDS (Non-Metric Dimensional Scaling). NMDS separates out lava cave mat communities at the phylum level, with *Proteobacteria* split out by class, from the overlying surface soils. Circles show the 95% confidence interval.

Non-metric dimensional scaling (NMDS) in Fig 6 shows a separation of the cave microbial mat samples from the surface soil samples, indicating that the two sets of samples are not similar to each other. The surface soil samples cluster tightly in the far right, while the cave samples spread out to the left. To fill out the dataset with one white sample per cave, we had LABE personnel obtain additional samples from GE-L350, Hopkins Chocolate, and S-L280 Caves in 2012. These three samples (GEM2, HC1, SC2) have very low numbers of OTUs and diversity compared to the other samples collected in 2011, although they have comparable overall sequence numbers to some of the original samples (e.g. GE2). Tan and yellow mats cluster together, while white mat samples are widely spread out and group into three clusters. Within cave similarity is greater than between cave similarity, probably due to the three mat colors sampled in each cave, and suggests the necessity for multiple samples to cover the diversity comprehensively.

### Differential abundance of OTUs between surface and cave

**Fig 7:**
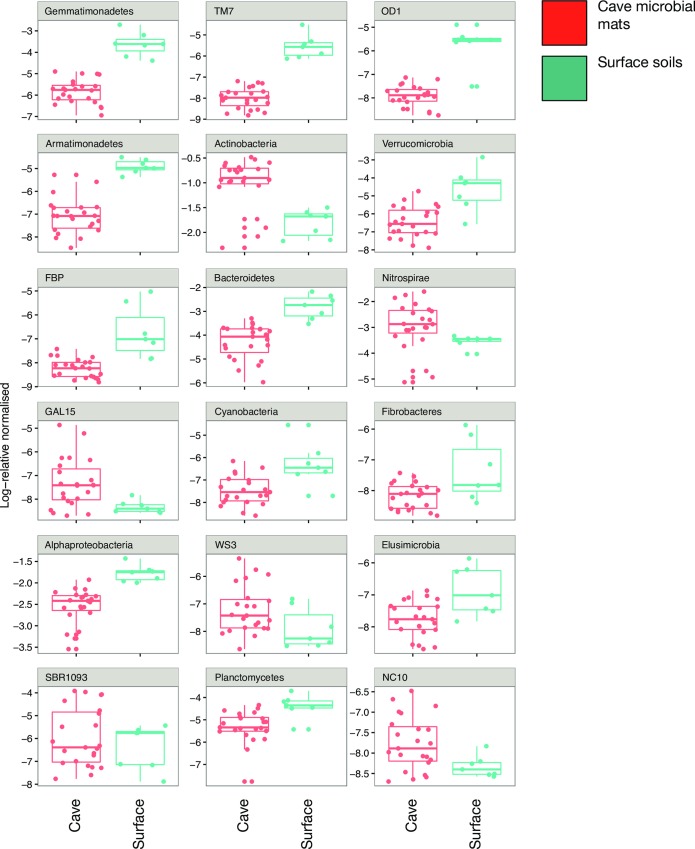
Plot of phyla and *Proteobacteria* class that were differentialy abundant between LABE surface soils and lava cave microbial mats. The band is the median, and the box delineates the upper and lower quartile. The whiskers show the maximum and minimum values. All data points are shown.

DESeq2 (differential analysis of count data) between all surface samples and all cave samples showed strong evidence for differential abundance of OTUs between surface soils and cave microbial mats (Fig 7). Eighteen phyla stood out as being significantly differential over or under represented between cave microbial mats and surface soils. The major phyla and *Proteobacteria* class that are significantly higher in the cave than in surface soil are the *Actinobacteria* (p>0.00000) and *Nitrospirae* (p = 0.00232). Major groups with greater representation in surface soils than the cave are *Gammaproteobacteria* (p>0.00000), *Verrucomicrobia* (p = 0.00001), *Bacteroidetes* (p = 0.00021), and *Alphaproteobacteria* (p = 0.02373). Of the minor phyla, *GAL15* (p = 0.00273), *WS3* (p = 0.03444), and *SBR1093* (p = 0.04227) have higher numbers in the cave samples compared to surface soils. The groups with higher amounts in surface soils than cave samples are *TM7* (p>0.00000), *OD1* (p>0.00000), *Armatimonadetes* (p>0.00000), FBP (p = 1e−05), *Cyanobacteria* p = 0.00438), *Fibrobacteres* (p = 0.00698), and *Elusimicrobia* (p = 0.04176). The remaining two minor phyla are approaching significant differences; *Planctomycetes* (p = 0.05177) and *NC10* (p = 0.05655).

### Scanning electron microscopy

**Fig 8:**
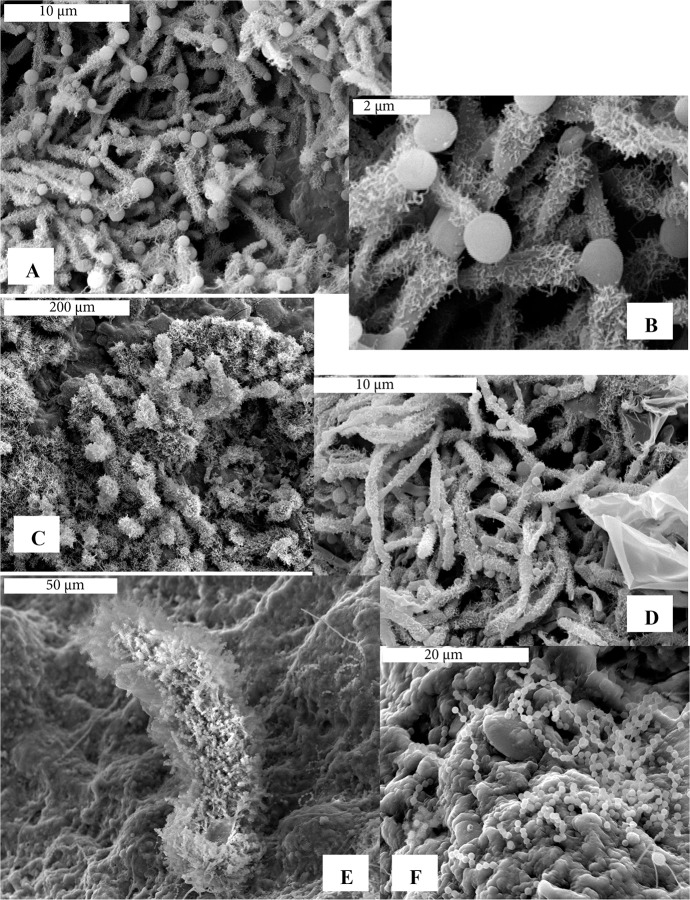
Scanning electron micrographs of LABE cave white and yellow microbial mats. (A) White microbial mat from Cave L-V460 showing filaments covered with putative pili/fimbrae with spheroid shapes emerging from the ends. (B) Close-up of these morphologies from A. (C) Overview of a field of these morphologies in the same white microbial mat from Cave L-V460. (D) Yellow microbial mat from Hopkins Chocolate Cave showing similar morphologies to those seen in images A-C, and including some biofilm and smooth filaments. (E) Overview of a yellow colony from Cave S-L280, showing extensive biofilm in the background and on colony edges. Beads-on-a-string morphologies are observed lying on the background biofilm. (F) Close-up of beads-on-a-string morphology on biofilm from Cave S-L280 yellow microbial mat.

The three samples (one white, two yellow) examined with scanning electron microscopy (SEM) revealed extensive microbial morphologies present with similarities and differences observed across the three samples. The white sample from Cave L-V460 contained the most unusual morphologies, which were filaments covered in curly putative pili/fimbrae with spheroids emerging from the tips (Fig 8A–8C). The spheroids range in size from 0.8 to 1.2 μm in diameter, and in Fig 8B, one can observe what appears to be a “neck” on one of the spheroids. Some of the filaments with numerous hair-like extensions appeared to be segmented (Fig 8B). Some parts of the sample also contained smooth, long filaments, while other areas had small spheroids (0.8 μm in diameter) emerging from the biofilm (not pictured). A somewhat similar morphology (filaments with spheroids protruding from the ends) was observed in a yellow sample from Hopkins Chocolate Cave (Fig 8D). This sample also had many colonies covered by, or partially emerging from, a lawn of biofilm (not pictured). The second yellow sample, from Cave S-L280, had extensive biofilm that appeared to bury filaments, and was dotted with large colony masses. We interpret the fuzzy areas along the margins of the colonies (Fig 8E) as being biofilm. Many strands of beads-on-a-string morphologies are seen on the colonies and biofilm (Fig 8F).

Energy dispersive X-ray spectroscopy revealed the presence of high silica and oxygen peaks, along with a carbon peak from the biofilm and microorganisms. The silica is precipitated as coatings on the basalt samples, inter-grown with biofilm and microorganisms.

### Cave environmental and geometry effects on richness

Several variables (Tables 1 and 2) collected from the field were tested to determine how much environmental conditions and cave geometry contribute to overall bacterial richness in the cave microbial mats. Temperature in the fall of 2012 was a positive predictor (41.1) of richness, while relative humidity in the fall was positive, but a small predictor of richness (6.2). Microbial mat color contribution to richness varied depending on color. Tan mats were a positive predictor (47.9). Yellow and white mats were negative predictors of richness (-11.5 and -35.1, respectively). Cave geometry variables were both positive and negative predictors. The number of entrances was a positive predictor (69.7). The complexity (nodes) of the cave was a negative predictor (-6.2). Caves with split (V-shaped) geometry were negative predictors (-20.2), while tube shaped was positive (21.9). With the exception of two caves (GD 20.6 and HC -19.8), caves were not predictive of overall richness. Neutral predictors (~ -3 to 3) were elevation of cave, overall length, distance from entrance, age of lava flow, and human impact.

## Discussion

The general belief for decades has been that cave microorganisms are a subset of the microbial community found in surface environments overlying the cave [50], eking out a minimal chemoheterotrophic existence in the cave. Our study is the first to test the hypothesis that bacterial communities in the soil overlying lava caves are substantially different from the bacterial communities found in the cave microbial mats. Our study is the most robust to date of lava cave microbial diversity, with surface soil and three colors of microbial mats (tan, white, and yellow) from each of seven different caves in Lava Beds National Monument, CA, USA.

### Cave and surface soil sample bacterial communities differ in major ways

Soil overlying caves can be a source of bacteria for colonization of underlying caves; however, the actual overlap in OTUs in our study is only 11.2%, suggesting that soil microorganisms either do not make it into the cave or die off once there. However, there are some similarities at bacterial higher taxonomic levels, i.e. phylum, between cave and surface, and between our soils and those of other regions. Both surface soils and cave microbial mats contain the phyla *Actinobacteria*, *Alpha-*, *Beta-*, *Delta-*, and *Gammaproteobacteria*, *Acidobacteria*, *Chloroflexi*, *Nitrospirae*, *Verrucomicrobia*, *Gemmatimonadetes*, *Planctomycetes*, and *Bacteroidetes*. These findings overlap with Janssen’s review [51] of studies of the microbial diversity of soils from many environments found in clone libraries. Across many habitats, these studies found that soils are dominated by *Proteobacteria*, averaging 39% of soil bacteria, and by *Acidobacteria*, *Actinobacteria*, *Chloroflexi*, *Verrucomicrobiota*, *Planctomycetes*, *Gemmatimonadetes*, *Bacteroidetes*, *Firmicutes*, and other/unknown. Our soils are fairly similar, but lack a significant amount of *Firmicutes*.

While not significantly different between cave and surface or among mat colors, *Acidobacteria* are a major phylum across all samples. Jones et al. [52] studied *Acidobacteria* across 87 soils and found them to be both ubiquitous and abundant. Their abundance relative to other taxa was variable, but correlated strongly with pH (R = -0.80, p<0.001). They suggest that pH is an effective habitat filter for *Acidobacteria*, with the greatest abundance below pH 5.5. Our pH values are from dripwater and pools and show no effect on bacterial diversity in LABE. Future studies will analyze pH of the rock substrate to determine if pH is a relevant controlling factor for diversity.

A study of Icelandic lava caves by Northup et al. [53] compared surface soil microbial communities with microbial communities of different types (mats, slimes, snottites, organic oozes, and soil) from four lava caves. Surface soils were the most diverse at two sites. As in our study, the mats were similar at the phylum level with *Actinobacteria* dominant followed by *Acidobacteria*, and *Alpha-*, *Beta-*, and *Gamma- Proteobacteria*. Unlike our study, there was a lack of *Nitrospirae* in the Icelandic caves. They found sample type to be the most important factor in bacterial diversity.

Ortiz et al. [25] collected surface soils from three locations above Kartchner Cavern. Analysis of their three soil samples was limited to qPCR for comparison of domain distributions within their karst cave speleothem swab samples. They found bacterial abundance in the cave was comparable to their soil samples, but *Archaea* were significantly higher in the cave, and fungi were below the detection limit in cave samples. Comparison of cave samples with one of the soil samples from above the cave showed an overlap of 16% of OTUs between cave and surface [24], which is comparable to our study of seven cave and surface soil samples, with an overlap of 11.12% OTUs. Our study and the Ortiz et al. [24,25] studies support the hypothesis that cave bacterial communities are significantly different from surface soil bacterial communities.

### *Actinobacteria* are more abundant in cave samples

A major difference between surface and cave bacterial communities in our study, and in comparison to the Ortiz et al. [24] study in a carbonate cave, is the abundance of *Actinobacteria*. While *Actinobacteria* occurred in moderate numbers in surface soils in our study (21%), they occurred at much higher levels in cave samples (39%). *Actinobacteria*, a dominant group in the 454 sequence libraries, are also probably some of the dominant forms seen in the scanning electron microscopy (e.g. Fig 8).

Barka et al. [54] reviewed *Actinobacteria* taxonomy, physiology, and secondary metabolite production. *Actinobacteria* are a large phylum among the Bacteria. Most *Actinobacteria* are heterotrophic, feeding on organic carbon, and some are known to fix nitrogen both as symbionts and free-living. New studies have also established that they can be chemolithoautotrophic, such as in the case of the uncultured TM3 subdivision of *Actinobacteria* that have been shown to exhibit nitrate-dependent iron oxidation [55]. Actinobacteria are key members of the microbial community in caves (e.g. [56]). *Actinobacteria* include organisms that give caves their musty odor due to the chemical geosmin [57]. Snider et al. [58] showed that *Actinobacteria* within two lava caves with roots penetrating the ceiling tended to be where more moisture is available, and their numbers fall off substantially in drier areas of the caves.

The color of the microbial mats may be produced by the bacteria present, in particular *Actinobacteria*. Production of melanoid pigments varies by strain, nutrients, and age [54]. Pigment colors range from red, yellow, orange, blue, green, and black [54]. Porca et al. [56] also reported that several *Pseudonocardiaceae* (relatives of their OTU group III) were shown to produce yellow pigments [59,60]. Lee [61] identified two new members of the *Pseudonocardiaceae* from soil and dry bat guano from a cave in the Republic of Korea that produced yellow and grey-white colored colonies.

Within the *Actinobacteria* recovered from our cave samples, the dominant organisms belonged to the family *Pseudonocardiacea*. Barton et al. [62,63] hypothesized that the *Pseudonocardiaceae*-related phylotypes found at one of their sites are degraders of plant matter coming into the caves. The *Pseudonocardiacea* are also known to produce a variety of secondary metabolites [64]. One note of caution is that universal bacterial primers are unable to amplify the 16S rRNA gene in many *Actinobacteria* despite 100% homology to the primers [65]. Thus, it is entirely likely that both our study and others are missing a portion of the *Actinobacteria* present in the environments sampled.

#### Broader implications of actinobacterial diversity in caves: The search for bioactive compounds

Caves are an extreme habitat and the cave microbiome has great potential as a novel resource for drug discovery [54,66,67]. Antimicrobials are one example of secondary metabolites produced by microorganisms, but may have broader roles as toxins, ionophores, bioregulators, and signal molecules produced by “metabolically talented” microbes [68]. Actinobacteria produce 2/3 of antibacterial agents in use, but are also important in biotechnology, medicine, and agriculture. A review of their extensive secondary metabolites by Barka et al. [54] includes antibacterials, anifungals, antivirals, antihelminths, antitumor, immunosuppressive and immunostimulatory agents, biopesticides, herbicides, and plant growth promoters. Natural products are the most promising source of novel antibiotics and cave environments have great potential for the development of new bioactive microbial metabolites especially from *Actinobacteria*, which are dominant in lava caves [69].

### Other bacteria of interest in lava cave microbial mats

In addition to the *Actinobacteria*, the gammaproteobacterial orders *Xanthomonadales* and unclassified *Gammaproteobacteria* were slightly elevated in cave samples (20%) in comparison to surface soil communities (18%). The order *Xanthomonadales* shows up in other cave studies on NCBI (i.e. accession numbers: DQ066611, FJ347998, HM592533). A poorly characterized taxon, *0319-6A21*, shows up with much greater abundance in the lava caves than in the surface soils. *0319-6A21* was first isolated in 2004 from Australian desert soil [70], and is classified in the class *Nitrospirales* and may provide clues into nitrogen cycling in caves. These results, together with the elevated *Pseudonocardiacea*, parallel the findings of Porca et al. [56] who found these groups to be major constituents of microbial communities in their studies of yellow colonies in carbonate caves in Spain, Slovenia, and the Czech Republic. Their study revealed three major core groups that included the *Pseudonocardiacea*, the *Chromatiales*, and the *Xanthomonadales*, which Porca et al. [56] suggest may be “true cave dwellers.”

An observation from the comparison of soil and cave samples at the genus level is the occurrence of many OTUs that were present in smaller numbers (genera that were present in three or fewer samples with less than 100 sequences), which we grouped together into the category minor genera. The genus level diversity in the minor genera in surface samples represents 35% of the diversity in surface soils samples and 20% of the diversity in cave samples.

One of the few lava cave microbial mat studies that extends the discussion of bacterial diversity to the level of genus is the Hawaiian study from 16 caves by Spilde et al. [71]. The samples included white and yellow microbial mats, along with pink-orange mats. At the genus level they reported an abundance of *Bacillus*, which were very rare in our LABE samples, *Nitrospira*, and two new genera of *Actinobacteria*: *Crossiella* and *Euzebya*.

### One bacterial community is different

Valentine 13 is not typical of either cave or surface samples. *Betaproteobacteria* (36.92%) dominate, while *Actinobacteria* make up only 8.56% of the total phyla. At the L6 genus level, only 1.44% of sequences are *Pseudonocardia*. Several variables may account for the differences. The site is located at a junction where the passage splits into two tubes. Very near the sample site is a large pile of breakdown, the only occurrence of breakdown of this size in the entire cave. The breakdown includes packed, wet sediment that has fallen through cracks in the ceiling above the pile, and the site is wetter than other locations in the cave. LABE personnel have observed millipedes and springtails around the edges of the breakdown, indicating a small connection with the surface. We speculate that the ceiling connection may be a source of microbes or nutrients unique to Valentine 13. The shape of the passage, with a higher ceiling at the collection site, may also direct more visitors to pass by the sample site, possibly also affecting the community structure or nutrient inputs. The floor also changes from pahoehoe to a’a lava at this point, perhaps indicating a change in rock chemistry. Regardless, we kept the sample in for analysis, and point out the need for multiple samples to mitigate such variations.

### Phylogenetic analysis of surface soils and cave microbial mats

**Fig 9:**
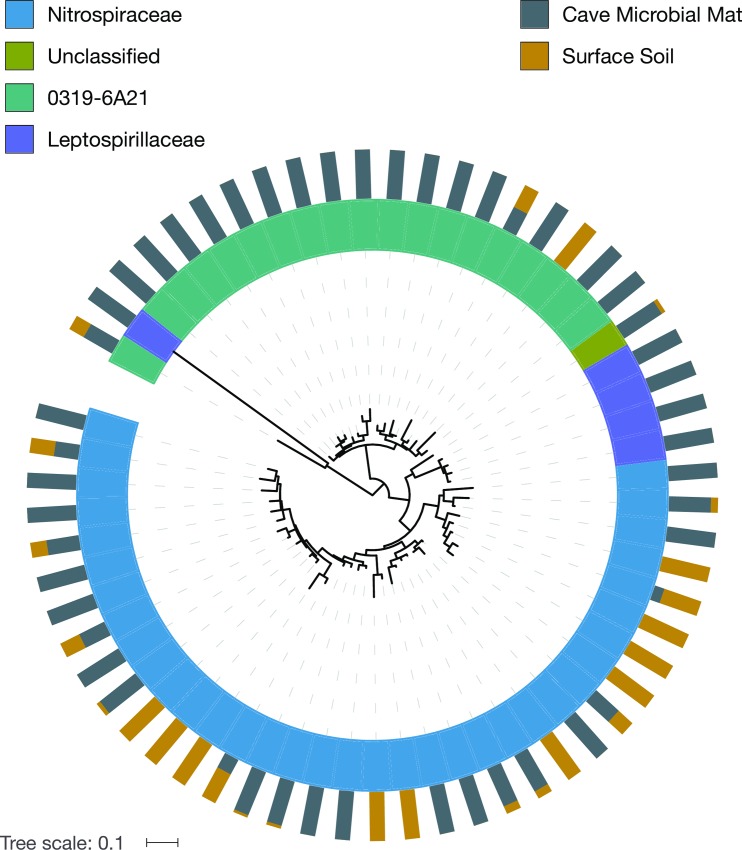
Phylogenetic tree of Nitrospirae by LABE lava cave and surface soil. Approximate maximum likelihood, mid-point rooted tree with pynast aligned 454 sequences.

The *Nitrospirae* phylum illustrates differences between OTUs found in cave samples versus surface soil samples. The phylogenetic tree of the *Nitrospirae* (Fig 9) shows the proportion of OTUs between cave and surface samples colored by family. The *Nitrospirae* vary by family in terms of whether they are primarily found in the cave vs. surface samples. The major *Nitrospirae* in our samples are found in three families, the *Nitrospiraceae*, the *Leptospirillaceae*, and a candidate family, *0319-6A21*. The majority of the *0319-6A21* family is found in the cave microbial mats and the remaining families both in the surface soil and the cave microbial mats. A similar tree was constructed for *Actinobacteria* (S2 Fig); however the proportion of cave taxa to surface taxa was nearly 50:50.

### Bacterial diversity among different colored mats

Bacterial composition was not substantially different among the mat colors, with some interesting differences. Members of the *Actinobacteria* dominated lava cave microbial mats of all colors. The *Actinobacteria* vary somewhat by mat color (S3 Fig), with 37% in tan mats, 39% in white mats, and 44% in yellow mats. *Gammaproteobacteria* varied the most by mat color with 19% in tan, 37% in white, and 21% in yellow.

Northup et al. [14] compared white and yellow microbial mats and selected secondary minerals based on color from four lava caves in each of three different locations; tropical and semi-arid lava caves in Hawaiˋi, temperate lava caves in the Azores, and semi-arid lava caves in New Mexico. Mats are more extensive in areas with greater rainfall. They found 13 phyla across all white and yellow mats from all three locations. The number of phyla per cave ranged from 5–11, with slightly greater diversity at the phylum level in yellow over white mats, but not in all phyla.

Hathaway et al. [21] was the first in-depth comparison of white and yellow microbial mat communities from lava caves on different archipelagos, but did not conclude that mat color was significant among differently colored mats. The authors concluded that geographic location is important in determining the composition of microbial communities, with Hawaiˋi showing greater diversity than Terceira. Principle Coordinate Analysis (PCoA) by island showed that only 13.7% of the variability was explained by geographic location and levels of nitrogen, organic carbon, and copper. Rainfall, especially in Hawaiˋi, accounted for another 10.8% of the variability. PCoA by cave showed a strong influence of geography. *Actinobacteria* (16%) dominated the clones recovered from Hawaiian samples, while on Terceira *Acidobacteria* dominated with 21% of the clones recovered. *Alphaproteobacteria* made up 13% of the diversity in Hawaiˋi and 15% in Terceira. Bacterial sequences recovered with no known phyla were 14% of the total in Hawaiˋi and 12% in Terceira. A nearest neighbor comparison among caves in Hawaiˋi and Terceira with other caves in the ARB database showed only thee OTUs (0.22%) overlap. This high level of alpha diversity means that bacterial communities found in each cave are different. These differences may be partially due to the greater range of habitats in Hawaiˋi, ranging from semi-arid to tropical rainforest conditions. Novel bacteria were found in all sites showing the need for conservation of caves as sources of novel bacteria and as simplified natural ecosystems for study of larger ecological questions.

Riquelme et al. [72] studied white, yellow, and tan mats from 14 lava caves on two islands in the Azores using 16S rDNA clone libraries. Environment and chemistry showed no relation to OTU diversity and composition of the microbial mats. Similar to other studies, including ours, there was a dominance of cosmopolitan OTUs. There was a greatest influence on β diversity (composition) between islands and caves than α diversity (community). The absence of clear differences across mat colors could be due to insufficient geologic time on these islands for microbial communities to diversify, and/or convergent evolution due to the selective pressure of extreme environments in caves.

We predict that a comparison of LABE microbial mats with those of the Azores, New Mexico, and Hawai`i, as new sequencing technologies become available for these sites, will show lower diversity in LABE than in Hawai`i in particular. Hathaway et al. [21] found that yellow and white color of the mat was not a predictive factor in diversity. Our findings in LABE were that yellow and white mat color was a negative predictor of diversity, but tan color was a strong positive predictor.

### Nitrogen cycling bacteria in LABE caves

Nitrogen is often a limiting nutrient in oligotrophic environments. Our cave sequences document the presence of several key organisms in the nitrogen cycle, some of which occur in elevated numbers in comparison to surface soil samples. We found *Nitrospirae*, which contain nitrite oxidizers and a *Nitrospira* spp. that can carry out complete nitrification, to be higher in cave samples (7%) in comparison to surface soil samples (3%). *Nitrospirae* OTUs varied from nine to 1064 across different caves and colored mats, with the highest number of OTUs occurring in tan mats from Golden Dome and GE-L350 Caves. The *Nitrospirae* contain nitrite oxidizers, the *Alphaproteobacteria* include nitrogen fixers, and *Betaproteobacteria* contain ammonia oxidizers, all key players in the nitrogen cycle. A new genus of *Nitrospira* has been described by Daims et al. [73] that can complete the entire nitrification cycle, taking ammonia to nitrate. Differences from cave to cave could be further investigated with a study of nitrogen available in the basaltic matrix and from infiltrating waters. Nitrite levels in our limited samples (Table 3) were largely low, but three caves had slightly elevated nitrate levels above the limit for drinking water of 1.0 ppm, and may indicate either surface inputs with nitrate or *in situ* production in these caves.

Other studies have suggested the importance of nitrogen-based systems in caves. Hathaway et al. [74] investigated the diversity of ammonia oxidation (*amoA*) and nitrogen fixation (*nifH*) genes in lava caves of Terceira, Azores, Portugal. They found that *Nitrosospirae* related sequences dominated the ammonia-oxidizing bacteria and that a key nitrogen fixation gene, *nifH*, was found among *Klebsiella pneumoniae*-like sequences (*Gammaproteobacteria*). Tetu et al. [75] found evidence that microbial slime curtain communities in the submerged Weebubbie Cave under the Nullarbor Plain in Australia had primary productivity based on the combined activity of ammonia-oxidizing *Archaea* and bacterial nitrite oxidizers, especially *Nitrospirae*. The ability of microorganisms to cycle nitrogen is key to supplying community needs in an oligotrophic environment. Future studies of LABE caves will employ metagenomics and transcriptomics to investigate the range of capabilities that LABE microorganisms possess and employ in the caves.

### Impact of human visitors on bacterial diversity

We divided our LABE study caves into high visitation (about 30,000 people a year) and low visitation, which have controlled access and are rarely visited by researchers and park personnel (up to 10 visitors some years). High and low visitation caves (Table 1), are comparable in terms of alpha diversity and show no significant differences in microbial community structure. When you make the jump to Carlsbad Cavern with over 400,000 visitors per year, Griffin et al. [76] reported significant human impacts of visitors on the microbiota. Using cultivation, they reported that *Staphylococcus* spp. were the dominant bacteria in the air along tourist trails compared to *Knoellia* spp. off trail. *Knoellia* is a new genus of *Actinobacteria* first isolated from a cave in China [77]. Fungal spores of *Penicillium* and *Aspergillus* showed a general decrease with distance from the entrance, but with a peak in the Lunchroom where visitors rest, eat, and wait for the elevator to return to the surface. A second study in Griffin et al. [76] using molecular techniques showed *Enterobacteriaceae* dominating along the descent rail and in the Lunchroom. They concluded that humans were important sources of non-indigenous microorganisms into Carlsbad Cavern, and recommended mitigation steps. There was only one *Staphylococcaeae* identified, and only 25 OTUs of *Enterbacteriacae* identified to the level of family detected at any LABE cave or surface sample. What we may be seeing is a threshold of visitors before we see human impacts. Right now, that threshold may be somewhere between 30,000 and 500,000 visitors per year, and merits further study.

### Environmental variables impact bacterial diversity differences

Levels of PO_4_^-^, SO_4_^2^, NO_3_^-^ in LABE lava caves were compared to those reported in lava caves from Terceira Island, Azores [74]. Phosphate levels in LABE samples are low, but much higher than in Terceira (ranges LABE 0.4697–0.6715 ppm vs. Terceira 0.003–0.0696 ppm), much lower in sulfate (ranges LABE 0.3251–1.1231 ppm vs. Terceira 1.42–37.29 ppm), and higher in nitrate (ranges LABE 0.5483–8.0036 ppm vs. Terceira 0.08–6.92 ppm). The nitrate results are unexpected given the large number of cattle roaming the surface above Terceira lava caves. Pools in Lechuguilla Cave [78], a carbonate cave in NM, had a more narrow range of nitrate values (ranges LABE 0.5483–8.0036 ppm vs. Lechuguilla 0.740–1.90 mg/L (ppm)) and a much greater range of sulfate values (ranges LABE 0.3251–1.1231 ppm vs Lechuguilla 28 to 112 mg/L (ppm)).

The strongest positive predictors of LABE bacterial diversity were the number of entrances to the cave and tan color. Tan mats also had the highest overall diversity. White and yellow colors were both negative predictors of diversity. Cave geometry varied as a predictor, with complex branching patterns (nodes) negative while a simple tube shape was a positive predictor of diversity. The cave itself had little effect on predicting diversity. All other variables were weak or neutral predictors of diversity, although temperature in Fall 2102 was a moderate predictor.

Cave geomicrobiological studies show that caves are nutrient-limited with redox interfaces in microniches where we see the interaction of microbial activities and minerals [79,80]. Interactions with minerals may be the dominant force driving microbial diversity in lava caves [81], but further study of basalt geochemistry at LABE is needed to test this hypothesis in the caves, but it seems likely that mineralogical factors do influence within cave diversity differences.

## Conclusions

Overlap in OTUs between surface and cave communities at LABE is only 11.12%, revealing that the cave bacterial communities differ substantially from surface soil communities and are not a subset of surface soils as previously assumed. Of particular significance are the differences in *Actinobacteria*, *Alphaproteobacteria*, *Nitrospirae*, and *Gammaproteobacteria* composition between surface soil and cave samples. Surface soil diversity is higher than that observed in cave samples and considerable novel diversity exists in both surface soil and cave mat samples. However, because many of the earlier studies are based on clone sequences that are limited in number, this conclusion may change as newer sequencing is applied to these sites. Communities in different mat colors do not differ substantially in composition, similar to the findings of Hathaway et al. [21]. A combination of cave geometry and environmental parameters influence microbial richness in lava caves.

LABE microbial cave community diversity at the phylum and *Proteobacteria* class level is comparable to diversity found in other major lava cave areas in New Mexico [8,10], Hawaiˋi [21,71], and the Azores [21,56,69]. Communities in different mat colors show significant overlap, but with many unique members that may contribute to the color of the mats in our study. So what does account for the differences in mat colors in lava caves? Caves are not homogeneous environments. There are zones related to distance from the entrance; seasonal variations; three-dimensional geometry; differential cooling and deposition of minerals during lava flows; and microhabitats that may vary at the level of the individual mineral grain. We think species composition and mineral availability at the microhabitat scale accounts for the color differences seen in the microbial mats.

Our study is the most extensive bacterial diversity study of lava caves to date that compares the bacterial diversity in three colors of microbial mats across seven caves with surface soil overlying each cave. Variability inherent in sampling supports the need for replicated study of microbial community structure. Further studies should examine diversity in other lava cave areas around the world using newer sequencing technologies. There are preliminary indications of world-wide microbial biogeography and we should work to fill in the gaps.

## Supporting information

S1 Fig Rarefaction curves by alpha diversity for microbial mats and surface soils. (PDF)

S2 Fig Phylogenetic tree of *Actinobacteria* by LABE lava cave and surface soil. Approximate maximum likelihood, mid-point rooted tree with pynast aligned 454 sequences. Outer bars show relative proportion of taxa found in cave microbial mats and surface soils. Inner band is colored by genus.
(PDF)

S3 Fig Cleveland dot plot of relative abundance of OTUs. Showing major and minor phyla for all cave and surface samples.
(PDF)
