# Supplementary figures and images for: Comparison of bacterial communities from lava cave microbial mats to overlying surface soils from Lava Beds National Monument, USA
Source: PLoS One. 2017 Feb 15;12(2):e0169339. doi: 10.1371/journal.pone.0169339 (PMC5310854; doi:10.1371/journal.pone.0169339)

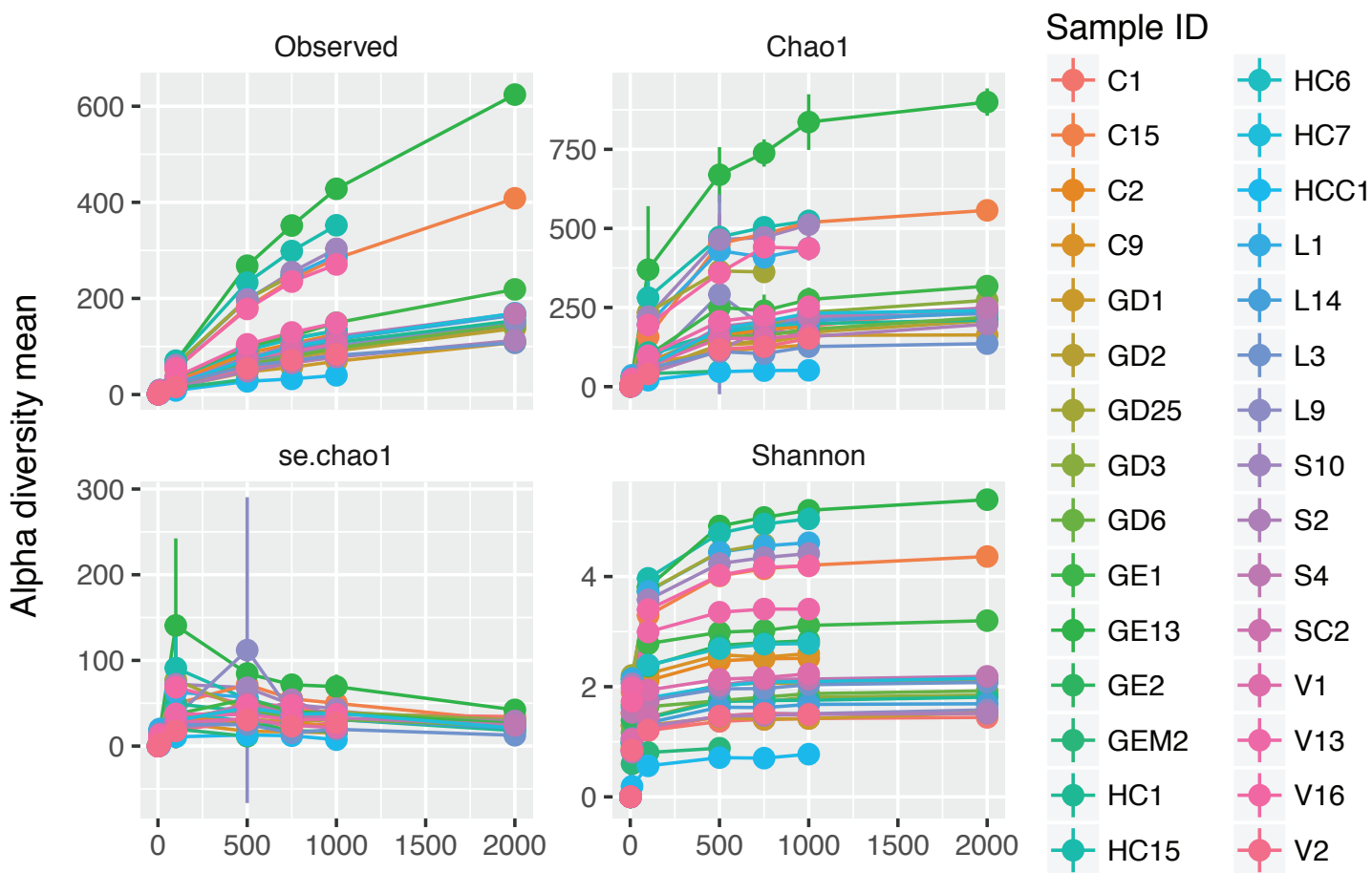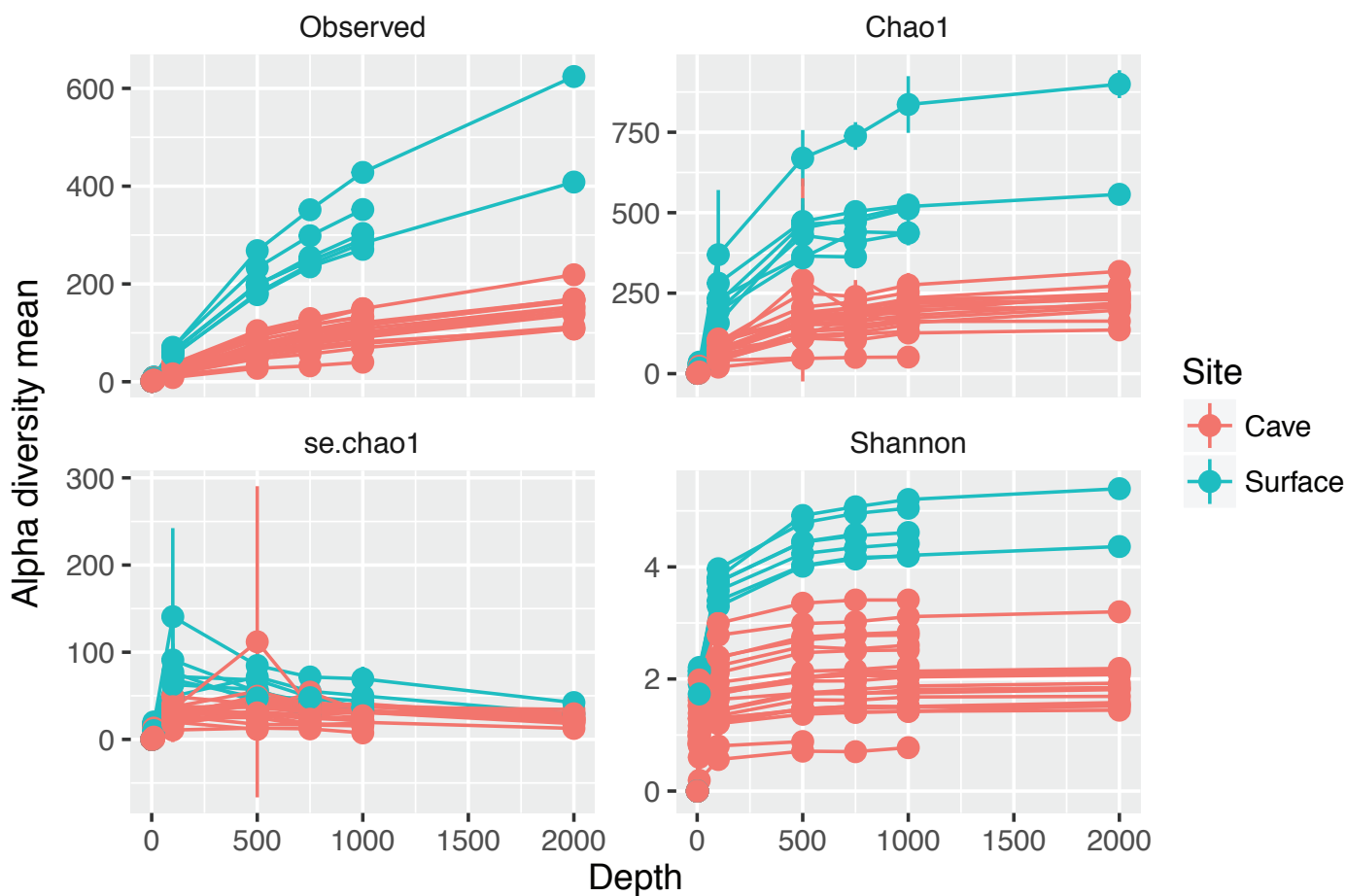

Supplement: S1 Fig — (PDF) [file pone.0169339.s001.pdf]

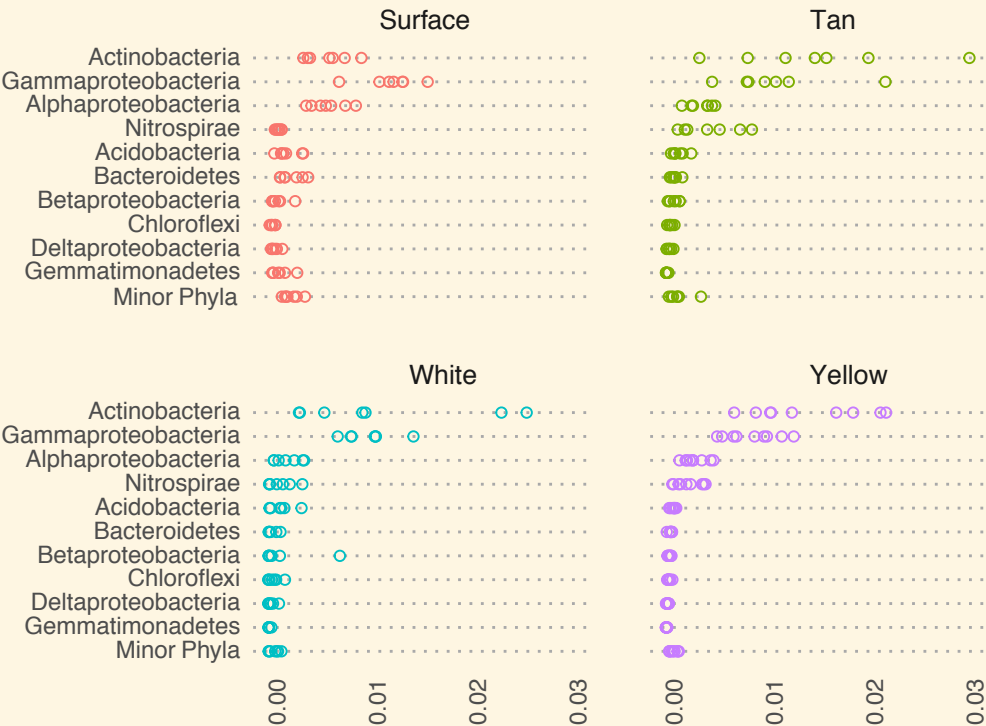

Supplement: S2 Fig — Approximate maximum likelihood, mid-point rooted tree with pynast aligned 454 sequences. Outer bars show relative proportion of taxa found in cave microbial mats and surface soils. Inner band is colored by genus. (PDF) [file pone.0169339.s002.pdf]

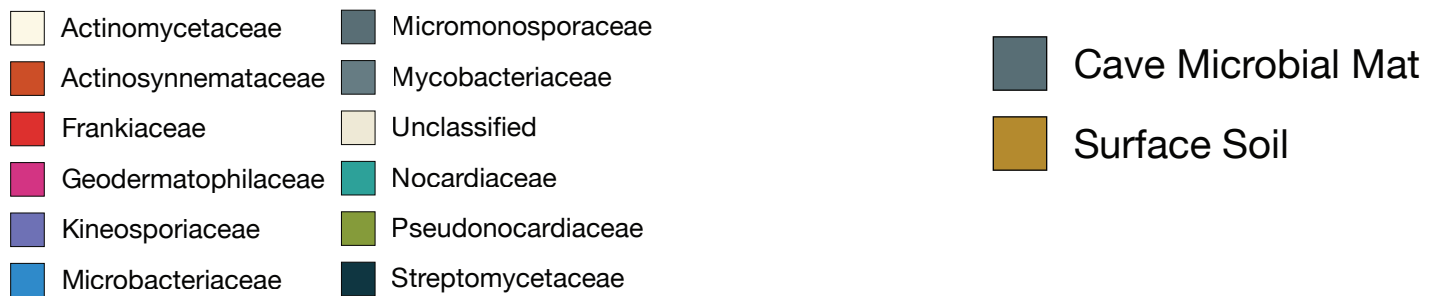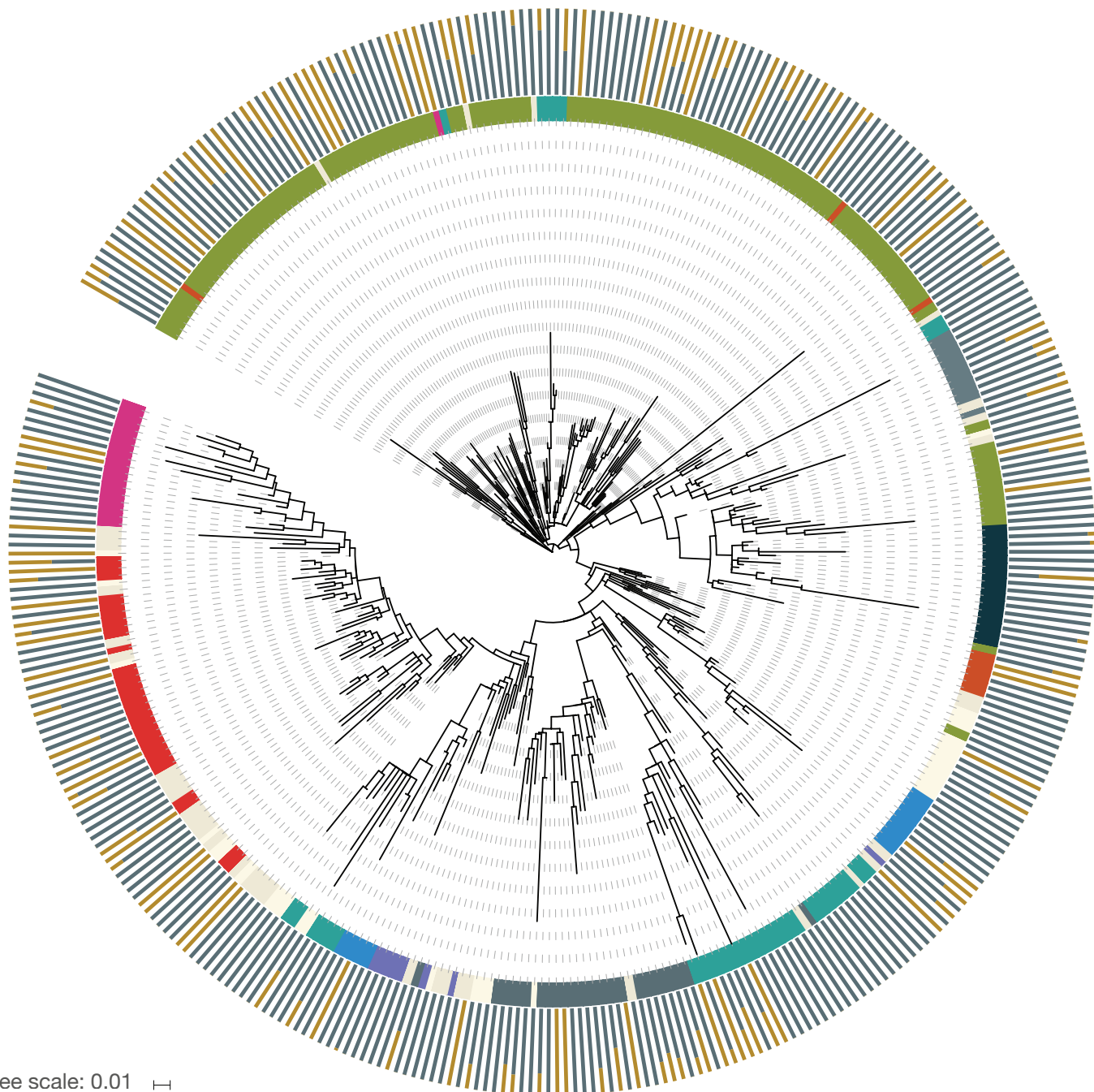

Tree scale: 0.01

Supplement: S3 Fig — Showing major and minor phyla for all cave and surface samples. (PDF) [file pone.0169339.s003.pdf]
